# Antioxidant Regulation and DNA Methylation Dynamics During *Mikania micrantha* Seed Germination Under Cold Stress

**DOI:** 10.3389/fpls.2022.856527

**Published:** 2022-04-08

**Authors:** Can Cui, Zhen Wang, Yingjuan Su, Ting Wang

**Affiliations:** ^1^School of Life Sciences, Sun Yat-sen University, Guangzhou, China; ^2^Research Institute of Sun Yat-sen University in Shenzhen, Shenzhen, China; ^3^College of Life Sciences, South China Agricultural University, Guangzhou, China

**Keywords:** *Mikania micrantha*, seed, cold, development, antioxidant, DNA methylation

## Abstract

As a primary goal, adaptation to cold climate could expand an invasion range of exotic plants. Here, we aimed to explore the regulation strategy of *M. micrantha* seed development under cold stress through molecular physiology and multi-omics analysis. Significant increase of hydrogen peroxide, malondialdehyde, and electrolyte leakage observed under cold stress revealed that oxidative damage within *M. micrantha* seed cells was induced in the initial germination phase. Proteomic data underscored an activation of antioxidant activity to maintain redox homeostasis, with a cluster of antioxidant proteins identified. Genomic-wide transcriptome, in combination with time-series whole-genome bisulfite sequencing mining, elucidated that seven candidate genes, which were the target of DNA demethylation-dependent ROS scavenging, were possibly associated with an *M. micrantha* germ break. Progressive gain of CHH context DNA methylation identified in an early germination phrase suggested a role of a DNA methylation pathway, while an active DNA demethylation pathway was also initiated during late seed development, which was in line with the expression trend of methylation and demethylation-related genes verified through qRT-PCR. These data pointed out that cold-dependent DNA demethylation and an antioxidant regulatory were involved together in restoring seed germination. The expression level of total 441 genes presented an opposite trend to the methylation divergence, while the expression of total 395 genes was proved to be negatively associated with their methylation levels. These data provided new insights into molecular reprograming events during *M. micrantha* seed development.

## Introduction

Seeds are considered as the crucial genetic delivery system essential for sustainable agriculture and landscape biodiversity. Successful germination is a critical step for plant establishment and the maintenance of natural ecosystems (Sawilska, 2007). The particular attributes of seed dispersal have important consequences for the evolution of ecological niches, i.e., the environment in which a species can persist (Van Den Elzen et al., 2016; Cordoba-Rodriguez et al., 2021). Long-range dispersal, which is usually assisted by wind, water, or animals, can significantly influence population dynamics as well as the evolutionary process of lineage diversification (Cain et al., 2000; Bohrer et al., 2005; Willis et al., 2014). In general, temperature, light, oxygen level, and soil moisture are the major factors determining the final seed germination rate (Ding et al., 2019). Among these factors, the temperature is the most crucial. Several studies have shown that the reproductive stage of the plant is the most sensitive to cold damage (Jia et al., 2015), and low temperature can directly inhibit the formation and elongation of functional roots at this stage (Ahamed et al., 2012; Wang J. et al., 2013; Zumajo-Cardona and Ambrose, 2020). To survive, plants respond to cold stress through a series of physiological and biochemical reactions controlled by a finely tuned regulatory network (Kenchanmane Raju et al., 2018). For instance, alterations in lipid composition, accumulation of compatible osmolytes, such as proline and sugars, and expression of cold-responsive genes occur in plants to adjust to the cold stress (Zhu et al., 2007; Urrutia et al., 2021). Most of the studies on seed functional ecology have mainly focused on the effect of seed dormancy and evolution on a plant genotype (Agrawal et al., 2021), the impact of seed dormancy on future evolutionary response to environmental change (Kazumi and Gerhard, 2021), or the influence of ecology and seed mass evolution on the timing of germination (Moles et al., 2005; Vandelook et al., 2018; Zumajo-Cardona and Ambrose, 2020); however, relatively less attention has been paid to the role of epigenetic regulation in seed germination, which may have evolved to optimize chances for seedling establishment.

*Mikania micrantha* H.B.K., a serious weed, is commonly called mile a minute, because of its rigorous and rampant growth performance. This invasive species has badly damaged agroforestry and tree crops in Southeast Asia (Waterhouse, 1994; Cock et al., 2000). Factors, such as comprehensive ecophysiological tolerance, rapid asexual expansion, allelopathic effects, and prolific seed production, have been hypothesized to facilitate the spread of *M. micrantha* (Shao et al., 2003; Song et al., 2007). Copious amounts of fine and fluffy seeds of *M. micrantha* can germinate in areas with suitable temperature and grow into new individuals in the following spring (Shao et al., 2005; Shen et al., 2007; Yu et al., 2009). Like most seed plants, a rapid and proper seedling establishment is very important for the invasion process of *M. micrantha*. In South China, *M. micrantha* flowers and achenes are formed between August and the following May, while peak seedling establishment generally occurs between September and November at the optimal temperature at 25–30°C (Yang et al., 2005). Although some seeds can germinate at 15 or 35°C, low/high ambient temperature could significantly inhibit *M. micrantha* seed development and seedling establishment (Yang et al., 2005; Macanawai et al., 2018). In South China, the temperature stays below 15°C during the winter (Yan et al., 2013). To cope with the low-winter temperature, *M. micrantha* plants accumulate anthocyanins, which turn the plant color from green to red (Zhang et al., 2019). Based on the samples of *M. micrantha* collected from Global Biodiversity Information Facility, National Plant Specimen Resource Center,^1^ National Specimen Information Infrastructure,^2^ and the China National Knowledge Infrastructure,^3^ *M. micrantha* has spread up to Fuzhou in Fujian Province, but no distribution point has been found in further north of China. Together, these pieces of evidence suggest that low-growth temperature is one of the main factors limiting the further spread of *M. micrantha* into northern China. To date, there has been little research on the influence of cold stress on *M. micrantha* seed development, and comprehensive analyses of molecular genetic variation and DNA methylation regulation during seed germination are especially lacking.

The low-temperature conditions can cause oxidative damage in plants by increasing a reactive oxygen species (ROS) level (Foyer et al., 2002; Gabriella et al., 2018). Once the temperature drops below the minimum temperature, irreversible damage to cells and tissues occurs, and seeds normally will not germinate (Krasensky and Jonak, 2012). To resist the cold stress, most plants have developed ROS scavenging systems, including antioxidants and antioxidant enzymes (Nonogaki et al., 2010). Scavengers, such as peroxidase (POD), catalase (CAT), superoxide dismutase (SOD), and ascorbate peroxidase (APX), can eliminate the over-accumulated ROS by activating the enzymatic antioxidant system, leading less damage to plant cells (Mittler et al., 2004; Raseetha et al., 2013). The cold condition may also induce changes at the cell level, such as osmotic stress, membrane modification, and low protein activity (Krasensky and Jonak, 2012). Malondialdehyde (MDA), as the main product of lipid peroxidation destructing the cell membrane induced by cold stress, is usually conducted as an indicator of a degree of oxidative damage (Girotti, 1985). The early stage of seed germination is usually accompanied by exosmosis of intracellular solutes (sugar, ions, amino acids, proteins, etc.), which is called imbibing damage (Bewley et al., 2013). Therefore, the conductivity of seed leaching solution will change accordingly. Moreover, proteomics and transcriptomics analyses have been conducted extensively to explore plant response to abiotic stress, including cold conditions (Bocian et al., 2015; Sun et al., 2016; Thomason et al., 2018). Up to now, proteomics analysis has confirmed the complexity of the response to cold-induced stress and identified main proteins involved in posttranslational modifications, signal transduction, lipid metabolism, inorganic ion transport, amino acid metabolism, and carbohydrate and energy metabolism on rice, *Arabidopsis thaliana*, *Thellungiellahalofila*, and other species (Cui et al., 2005; Gao et al., 2009; Gammulla et al., 2010; Wang et al., 2016). Abundant genes that responded to cold stress have been documented from diverse plants, which were called as COR (cold regulated), KIN (cold-induced), LTI (low-temperature-induced), or RD (responsible to dehydration) genes (Nordin et al., 1993; Welin et al., 1995; Medina et al., 1999).

Although the physiological and molecular changes that occur under cold conditions have been extensively reported in economically important crops (such as rice and maize), the endogenous factors contributing to cold stress tolerance in invasive plant species remain unclear (Yan et al., 2006; Ji et al., 2017; Urrutia et al., 2021). DNA methylation is an important epigenetic modification and is closely related to the ecological adaptation of plants (Strahl and Allis, 2000; Geiman and Robertson, 2002; Chinnusamy and Zhu, 2009). It has been well documented that DNA methylation can function as an epigenetic regulator to potentially provide flexible genomic parameters for plants to adjust to cold stress (Chinnusamy and Zhu, 2009; Huff and Zilberman, 2012; Sahu et al., 2013; Thiebaut et al., 2019; Wang et al., 2019a). In plants, DNA methylation mainly occurs in three different sequence contexts: CG (symmetric), CHG (symmetric), and CHH (asymmetric); H refers to any nucleotide, except guanine (Feng et al., 2020). There has been a growing body of literature confirming that the global loss of methylation in the CG context results in improper germ development, poor or no-seed viability in *Arabidopsis*, and severe necrotic lesions in rice (Dubin et al., 2015; Xie et al., 2020). Loss of DNA methylation in CHG and CHH contexts, reportedly, does not affect seed development and viability in *Arabidopsis* (Dubin et al., 2015). However, little is known about how DNA methylation regulates the germination of *M. micrantha* seeds under cold stress.

In this study, we used an integrated approach involving physiological characterization, data-independent acquisition mass spectrometry (DIA-MS), RNA-Seq, and single-based-resolution DNA methylome profiling of *M. micrantha* seeds at successive embryonic stages. Additionally, genes potentially involved in ROS scavenging and DNA methylation during seed germination under cold stress were also determined. To the best of our knowledge, this is the first report to examine both the function of molecular response mechanisms and the epigenetic control of seed germination induced by cold stress in *M. micrantha*. Our results provide valuable insights into the role of key regulatory factors and DNA methylation in *M. micrantha* seed development.

## Materials and Methods

### Plant Material and Seed Germination Assay

Seeds were collected at maturity from *M. micrantha* grown at Neilingding island (Shenzhen, Guangdong, China), and were stored after harvest at 20°C until dormancy was fully alleviated. Then, seeds were surface sterilized with 75% alcohol for 5 min, followed by rinsing five times with sterile-deionized water. Then seeds were surface-sterilized with 75% alcohol for 5 min and 20% sodium hypochlorite solution for 30 min, both followed by rinsing five times with sterile deionized water. Each of 30 grains dried with sterilized filter paper was placed in the Petri dishes (90 mm in diameter) with a Murashige and Skoog medium (MS), containing 0.443% MS, 3% sucrose, and 0.75% agar. Seeds were grown in a chamber (14, 25, and 30°C; 1,000 μmol photons/m^2^ s photosynthetically active radiation; 16-h day/8-h night; 40–60% relative humidity) for 2 weeks. Seed germination was defined by the protrusion of the radicle (when the radicle had pierced the envelopes with an observed radicle emergence of 0.5 cm) and the germination counts were made daily for 14 days. For the soil-growth experiment, the experimental design consisted in the same condition as the MS medium performed. A total of 300 seeds were sown in three soil pots containing substrate soil vermiculite (1:1), each pot containing 100 seeds. The corresponding data, including germination rate, length of germ, generation of hydrogen peroxide (H_2_O_2_) and MDA, and electrolyte leakage, were recorded. Three successive experiments were necessary to obtain valid data from three biological replicates.

**FIGURE 1:**
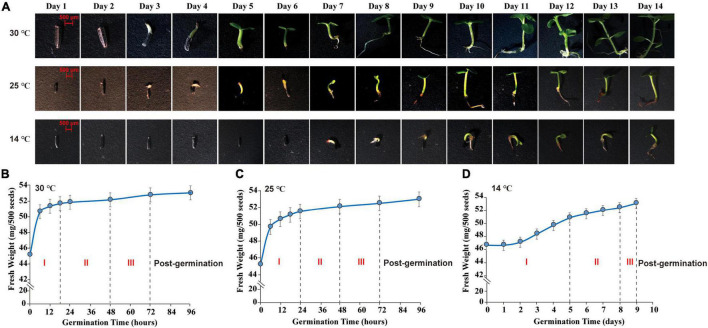
Morphological observation and water absorption analysis of *M. micrantha* seed germination. **(A)** Effect of temperature on morphology of 2-week *M. micrantha* seedling. Time course of water absorption in germinating *M. micrantha* seed at 30°C **(B)**, 25°C **(C)**, and 14°C **(D)**.

Seed morphological features were surveyed and photographed by a stereomicroscope (SteREOLumar. V12, ZEISS, Germany). The germ of *M. micrantha* seeds emerged on the 3^rd^ day, reaching a length of 0.3–1.0 cm at 25 and 30°C (Figure 1A). The emergence of germs indicated the completion of seed germination and the beginning of seedling growth (Nonogaki et al., 2010; Weitbrecht et al., 2011). However, the hypocotyl emerged through the seed coat on the 7^th^ day, with visible elongation at 14°C, indicating that seed imbibition was delayed under the cold condition. A water uptake curve was constructed by recording the change in seed fresh weight during germination (Figures 1B–D). *Mikania micrantha* seeds absorbed water rapidly from 0 to 18 h of germination at 30°C, and the fresh weight of seeds increased faster in the imbibition stage (imbibition-Phase I). After 18 h, the rate of increase in seed weight decreased, resulting in a plateau in water uptake (protrusion-Phase II). Approximately, 40% of the seed germ had emerged through the seed coat at 48 h, and germ growth continued until 72 h, which marked the end of germination (germination-Phase III). Thus, Days 3 and 7 were selected as the sampling time points to represent the termination of *M. micrantha* seed germination and the start of seedling growth, respectively. The different seed germination stages showed large differences under cold stress. The time of Phase I was delayed to 24 h at 25°C (Figure 1C). However, seeds completed the first stage of water uptake (Phase I) and entered the plateau stage (Phase II) until the 5^th^ day at 14°C. The germ of only 25% of the seeds broke through the seed coat (Phase III) on the 8^th^ day, and seedling growth and development started on the 9^th^ day (Figure 1D). Correspondingly, Days 7 and 10 were chosen as the sampling time points, mainly because the germ length of seeds subjected to 14°C at these time points was equal to that on Days 3 and 7, respectively, at 25 or 30°C.

Samples collected from 3^rd^ to 7^th^ days (25 and 30°C), 7^th^ day and 10^th^ day (14°C) after imbibition were washed gently with sterile water, and germinated germs from 3^rd^ day at 25°C and 30°C, 7^th^ day at 14°C were peeled off the seed coat on the ice for the subsequent analysis. One part was snapfrozen with liquid nitrogen and stored at –80°C for the DIA-based proteome and enzyme activity analysis, another part was stored at an RNA fixer (BioTeke, Shanghai, China) at –20°C for subsequent RNA-Seq analysis. For the content determination of MDA, about 0.1-g germs were grounded with 5 ml of a 0.05-M sodium phosphate buffer, followed by centrifugation at 10,000 × *g* for 15 min. The supernatant solution obtained after centrifugation was used for determining MDA content according to the method as described by Wang J. et al. (2013). Total H_2_O_2_ content was determined with spectrophotometry according to Patterson et al. (1984). The electrolyte leakage of seed leachate was measured by the conductivity meter (DDS-307A, INESA INSTRUMENT, Shanghai, China).

### RNA-Seq and DIA-Based Proteomics Analysis

Methods of RNA extraction, construction of cDNA library, transcriptome sequencing, protein extraction, desalting and digestion, DDA/DIA-MS data analysis, protein functional annotation, differential expression analysis of proteins (DEPs), differential expression analysis of genes (DEGs), and data analysis were shown as Supplementary Information, which were the same as reported in our previous study (Cui et al., 2021).

### Whole-Genome Bisulfite Sequencing, Methylation Calling, and Identification of Differentially Methylated Regions and Differentially Methylated Region-Associated Genes

Genomic DNA was extracted using a TIANamp genomic DNA kit (DP305) based on the manufacturer’s procedure. Agarose gels were performed to validate the degradation and contamination of the genomic DNA. Total 100-ng high-quality DNA spiked with 0.5-g lambda DNA was fragmented into a mean size of 200–300 bp *via* sonication (Covaris S220, Massachusetts, United States). True Seq-methylated adapters were ligated to the end of fragmented DNA by treating with a bisulfite using EZ DNA Methylation-Gold™ Kit (Zymo Research Corporation, CA, United States). The whole-genome bisulfite sequencing libraries were constructed and evaluated by an Agilent Bioanalyzer 2100 system. Image analysis and base calling were performed with an Illumina CASAVA pipeline. Sequencing in a paired-end mode on the Illumina platform (Illumina, CA, United States) generated 150-bp long reads at 30 × sequencing depth of an *M. micrantha* genome. Three biological replicates were sequenced for each stage of seed development.

Raw reads generated from the Illumina pipeline in FASTQ format were pre-processed to remove low-quality bases, adaptor sequences with fast p. 0.20.0. Clean reads after filter were aligned with the chromosome-level *M. micrantha* reference genome using Bismark (v0.16.3), and only reads mapped at the unique position were retained for methylation calling (Andrews, 2011). Naturally, unmethylated lambda DNA was added to samples to estimate the efficiency of bisulfite conversion and an error rate. The methylated cytosines (mC) were extracted with deduplicates after a pre-deduplication step as recommended by Bismark (v0.16.3). The DNA methylation level at each *methylcytosine* site or region was determined by percentage of reads supporting mC to the total C and T at the same site (Garg et al., 2015; Bhatia et al., 2018; Rajkumar et al., 2020). *Q*-value for each methylated site was calculated using the binomial test with methylated counts (mC). The true methylated sites were selected with *Q*-value < 0.05 and sequencing depth ≥ 5 (Garg et al., 2015; Bhatia et al., 2018).

A methylation level in genomic regions, including promoter, exon, intron, repeat, and 2 kb flanking regions, was determined using Perl scripts. The methylation levels in the gene body and flanking regions (2 kb of an upstream or downstream region) were determined by partitioning the sequence into 100 bins of equal size and evaluated as a weighted methylation level. Bioconductor package DSS was performed for calling differentially methylated regions (DMRs) between different stages under three cultivated temperatures based on the binomial test *Q*-values. DMRs were called for exceeding a minimum length (100 bp slide windows by default) and cover more than a minimum number of three CpG sites with *Q*-values less than 0.05 (Feng et al., 2014; Wu et al., 2015; Park and Wu, 2016). DMR-associated genes were defined as genes that overlapped DMRs within 2-kb flanking regions or genic regions for each comparison. DMRs located across flanking regions and gene body regions were considered in both categories. GO functional enrichment analysis of genes associated with hyper and hypomethylated DMRs was carried out using R package-GOseq; GO terms with corrected *Q*-values less than 0.05 were selected as significantly enriched (Young et al., 2010). The statistical enrichment of KEGG pathways of DMRs-associated genes were conducted by KOBAS (Mao et al., 2005).

### Antioxidant Enzyme Activity Detection

Activities of various antioxidant enzymes were determined as described by Rao et al. (1996) with little modification. The activity of SOD was analyzed by measuring ferricytochrome *c* reduction at 560 nm. Specifically, one unit of SOD was defined as the amount of an enzyme that inhibited the rate of ferricytochrome *c* reduction by 50%. The activity of CAT was determined according to the consumption of H_2_O_2_ at 240 nm for 2 min. The activity of POD was assayed with a reaction mixture containing a 2.7-ml phosphate buffer (pH 7.0), 2-mM EDTA, 300-mM H_2_O_2_, and 100-μL enzyme supernatant. POD activity was determined by the increase in the absorbance at 470 nm for 1 min due to guaiacol oxidation (Maehly, 1954). For the soil-growth experiment, a total of 300 seeds were sown in three soil pots containing substrate soilvermiculite (1:1), each pot containing 100 seeds. The corresponding data, including germination rate, length of germ, generation of hydrogen peroxide (H_2_O_2_) and MDA, and electrolyte leakage, were recorded and calculated.

### Identification of DNA Methyltransferase and Demethylase Genes and Quantitative Real-Time PCR Validation (qRT-PCR)

Protein sequences of *A. thaliana* methyltransferase and demethylase were obtained from https://www.arabidopsis.org. By running BLASTP, the amino acid sequences collected from *A. thaliana* were used as seed sequences to detect all DNA methylation-related genes encoding DNA methyltransferases and demethylases based on the *M. micrantha* genome database (Altschul et al., 1997). Subsequently, the best hits of amino acid sequences were selected as candidates. These genes were randomly selected to be quantified with qRT-PCR.

First-strand cDNA of all samples was synthesized using Evo M-MLV RT Master Mix (Accurate Biology, Guangzhou, China). Quantitative RT-PCR was performed with ChamQ SYBR^§^ qPCR Master Mix by Vazyme and carried out in the Roche LightCycler480 II detection system (Roche, Switzerland). Each amplification was performed in a 10 μL reaction mixture, containing 5 μL of ChamQ SYBR Mix (2 ×, Vazyme, Nanjing, China), 0.4 μL of a primer pair, 1 μL of cDNA, and 3.6-μL of ddH_2_O. The reaction procedures for qRT-PCR were as follows: 95°C for 30 s (preincubation), 40 cycles of 95°C for 10 s, 60°C for 30 s, and 72°C for 20 s (amplication), followed by 95°C for 15 s, 60°C for 1 min and 95°C for 15 s (a melting curve), and then cooling at 40°C for 1 min. Primers used for qRT-PCR were designed by Primer3 tools according to cDNA sequences and synthesized by Tsingke Biotech (Supplementary Table 1). These primers had been proved to be able to amplify the target genes specifically (Supplementary Figure 1). The housekeeping gene β-actin was used as the standard internal control, and the relative expression level of each gene was calculated using the 2^–ΔΔ^ *^Ct^* method (Livak and Schmittgen, 2001; Lo Piero et al., 2005; Lin and Lai, 2010). The relative quantitation of gene expression of the structural genes involved in DNA methylation (*MmSHH1*, *MmAGO6*, *MmRRP6L1*, *MmMORC1*, *MmIDN2*, *MmNRPE9B*, *MmNRPE1*, *MmDDM1*, *MmHDA6*, *MmUBP26*, *MmDNMT1*, *MmCMT2*, *MmCMT3*, *MmDME*, and *MmROS1*) and in the regulation of the redox process (*MmCAT1*, *MmCAT2*, *MmCAT3*, *MmCAT4*, *MmSOD1*, *MmSOD2*, *MmSOD3*, *MmSOD4*, *MmPOD1*, *MmPOD2*, *MmPOD3*, and *MmPOD4*), each gene was conducted in triplicate. Negative controls without reverse transcriptase were routinely included.

### Data Analysis

Statistical significance in all texts was carried out at the *Q*-value = 0.05 level using SPSS (version 13.0, SPSS Inc., Chicago, IL., United States); All the significant differences were analyzed by One-way ANOVA; principal components analysis (PCA) was conducted with basic function prcomp in R package; genome-wide profiles of DNA methylation in circos were performed with TB tools; The box plots were plotted by Python 3.9; heatmaps and violin were generated by gplots and vioplot in R package.

## Results

### Morphological and Physiological Characterization of *Mikania micrantha* Seeds

**FIGURE 2:**
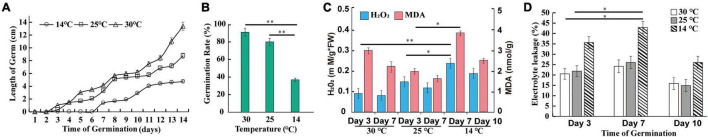
Effect of temperature on *M. micrantha* seed germination at 14, 25, and 30°C. Germ length **(A)**, germination rate **(B)**, hydrogen peroxide and MDA contents **(C)**, and electrolyte leakage **(D)** of seed leachate at three key time points, covering *M. micrantha* seeds developmental stages. One-way ANOVA, *p* ≤ 0.05 (*), *p* ≤ 0.01 (**).

As shown in Figure 2A, there was no significant difference between germ length at 30°C and 25°C until the 4th day. Furthermore, the cold condition affected the seed germination rate. As illustrated in Figure 2B, the germination ability was approximately 80% at 25°C and slightly, but not statistically significantly, higher at 30°C. Compared with 25°C, the seed germination rate at 14°C was lower by 54.25%, a significant difference (*Q-*value = 1.98 × 10^–4^). To further investigate the temperature-dependent germination behaviors, we analyzed the ROS content of *M. micrantha* germs, since the low temperature has been shown to induce ROS accumulation in germs during early germination (Petrov et al., 2015). The content of hydrogen peroxide (H_2_O_2_), one of the main constituent substances components of ROS, was significantly rich at 7^th^ day of 14°C (Figure 2C), but lower when the H_2_O_2_ content on the 3^rd^ day is decreased to 48.81 and 57.58% at 25°C (48.81%) and 30°C (57.58%), respectively. As shown in Figure 2D, the content of MDA significantly increased in germs at 7^th^ day of 14°C compared to the 3^rd^ day at 25°C and 30°C. Furthermore, changes in electrical conductivity, as indicated by the electrolyte leakage, at 30°C were generally consistent with those at 14 and 25°C (Figure 2D). At the early germination stage, the slight increase in conductivity demonstrated that the cell membrane suffered serious damage. After the 7^th^ day, the conductivity gradually decreased, possibly because the damaged cell membrane was gradually relieved.

### Response to Cold Stress of Seed Germination Cultivated in Soil Was Similar to That in Culture Medium

To compare the effect of cold stress in *M. micrantha* seed germination cultivated in soil to those observed in the MS culture medium, same seeds sown at three temperatures were performed to observe morphological features. As indicated in Supplementary Figure 2A, the germination rate of seeds and seedling establishment showed almost no change between incubation at 30 and 25°C during the 14 days of growth. The seeds started to germinate from the 6^th^ day at 14°C, which was slightly earlier compared to those incubated in the MS medium (Supplementary Figure 2B). And the radicle growth was obviously slowed down in response to cold stress at 14°C. In addition, there was no significant difference in the germs growth rate between 25 and 30°C. As shown in Supplementary Figure 2B, the radicle length reached 12.62 cm and 13.61 cm under 25 and 30°C culture conditions at the 14^th^ day, respectively, while seed germination was significantly inhibited at 14°C by about 71% compared to 25°C (*Q*-value = 0.0184) as shown in Supplementary Figure 2C. The seed germination status, seedling growth under the MS medium, and soil culture remained basically the same (Supplementary Figures 2D,E).

### Changes in *Mikania micrantha* Germ Transcript Profiles Under Cold Stress

**FIGURE 3:**
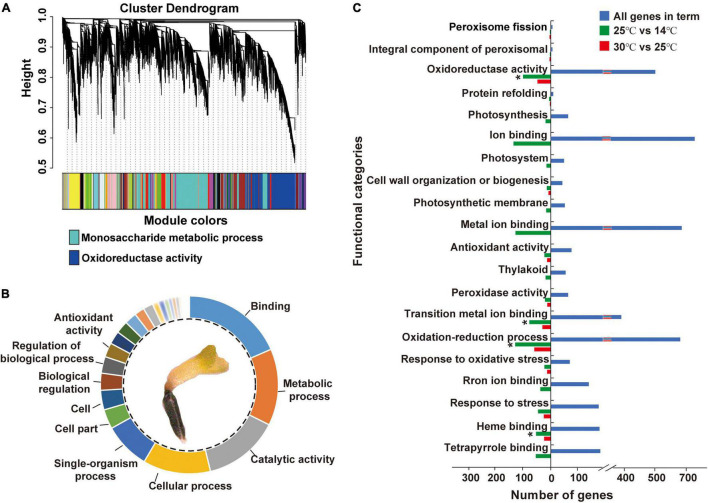
Differentially expressed genes (DEGs) and their gene ontology (GO) annotations during the imbibition of *M. micrantha* seed. **(A)** Hierarchical cluster analysis of genes from the *M. micrantha* transcriptome. The co-expression clusters are assigned as different colors. **(B)** Functional distributions of gene ontology (GO) annotation in germinated germs of all DEGs. **(C)** Function categories of DEGs in the 30°C vs. 25°C, and 25°C vs. 14°C comparisons. One-way ANOVA, *p* ≤ 0.05 (*).

Approximately, 78,275,481 raw sequence reads were generated *via* RNA-Seq, of which 96.82% were retained as clean reads after the removal of low-quality reads. A total of 40,088 genes were confirmed from the library with an average mapping rate of 90.31% to an *M. micrantha* reference genome (Supplementary Table 2). Gene co-expression analysis modules showed a relatively consistent expression pattern. According to the results of cluster analysis, the dark-turquoise module contained the most genes, followed by the midnight-blue module (Figure 3A). Pathway enrichment analysis of gene modules showed that the dark-turquoise module genes were mainly enriched in the monosaccharide metabolic process, while the midnight-blue module genes were mainly involved in oxidoreductase activity. Gene ontology (GO) functional analysis indicated that binding, the metabolic process, catalytic activity, the cellular process, the single-organism process, and antioxidant activity-related genes were moderately significantly overrepresented (Figure 3B). Genes responsible for antioxidant activity accounted for 2.59% of all identified genes, indicating that cold treatment stimulates antioxidant activities during *M. micrantha* seed development. Approximately, 1,116 genes were identified as statistically differentially expressed genes (DEGs) based on the criteria of twofold increase or decrease in expression [-1 ≥ log(FC) ≥ 1] (Figure 3C). Enrichment analysis of all DEGs in functional categories reflected some of these abundantly represented genes: ion binding, the oxidation-reduction process, metal ion binding, oxidoreductase activity, tetrapyrrole binding, and response to stress. These categories included genes encoding copper chaperones for superoxide dismutase (SOD) and genes implicated in redox, like primary-amine oxidase and adenylyl-sulfate reductase activities, which indicated that most of the changes in gene expression were strongly induced by cold stress at 14°C.

### Cold Stress Response in the Proteome of Germinating *Mikania micrantha* Seeds

**FIGURE 4:**
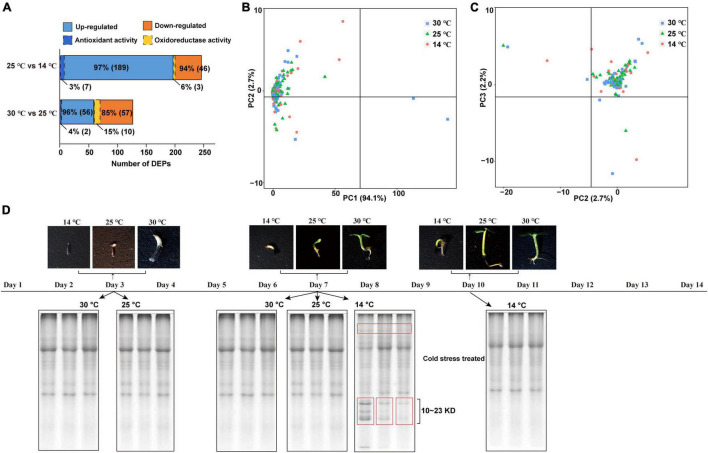
Proteins expression profiles of *M. micrantha* germs. **(A)** Numbers of differentially regulated DEPs, and number of antioxidant/oxidoreductase-related DEPs identified from the comparison groups of 25°C and 14°C, 30°C and 25°C. **(B,C)** Principal component analysis of proteins identified from germs germinated at 14°C, 25°C, and 30°C. A PC1- × -PC2 plan **(B)**; a PC2- × -PC3 plan **(C)**. **(D)** SDS-PAGE analysis of soluble proteins isolated from 3^rd^ and 7^th^ days at 25°C and 30°C, and 7^th^ and 10^th^ days at 14°C.

A total of 8,334 proteins were identified *via* DIA-based proteome analysis, of which 3,631 (43.57%) were significantly affected by the cold condition (one-way ANOVA, *Q*-value < 0.05). As shown in Figure 4A, the abundance of 49 proteins significantly increased at 14°C (FC ≥ 2.) compared with 25°C. Among these, three proteins related to oxidoreductase activity were statistically highly expressed in response to cold acclimation (Supplementary Table 3). On the other hand, 196 proteins showed lower abundance at 14°C than at 25°C, and 7 of these proteins related to antioxidant activity. The level of 58 proteins was significantly higher, and that of 67 proteins was significantly lower at 25°C than at 30°C; among these, 2 and 10 proteins, respectively, were related to oxidoreductase activity. Principal component analysis (PCA) showed that proteins identified at 14°C were relatively more dispersed than those identified at 25 and 30°C, indicating that proteome was more strongly affected by the decrease in temperature from 25 to 14°C than by that from 30 to 25°C (Figures 4B,C). Additionally, PC1 explained 94.1% of the variance. To separate the soluble proteins based on molecular weight, we performed sodium dodecyl sulfate–polyacrylamide gel electrophoresis (SDS–PAGE) (Figure 4D). The results showed that samples treated with cold stress contained a greater abundance of proteins with molecular weight, ranging from 10 to 23 kDa, which suggests that more protein species were induced at 14°C to facilitate plant adaptation to the cold condition.

### Analysis of the Antioxidant Pathway Under Cold Stress

**FIGURE 5:**
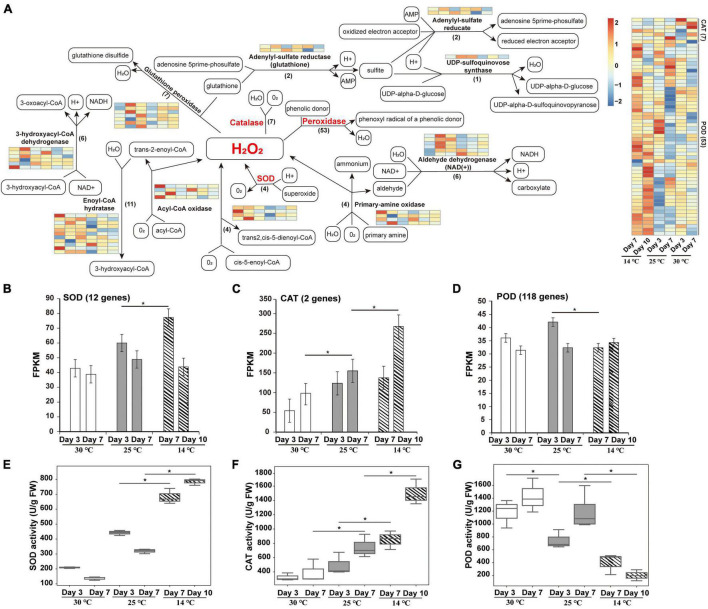
Redox pathway enrichment analysis of DEPs identified during the germination of an *M. micrantha* seed at 14, 25, and 30°C. **(A)** Redox pathway connected by a black straight arrow line/a black dashed arrow line. Relative levels of DEPs expression are shown by a color gradient from low (blue) to high (red). **(B–D)** Fragments per kilobase of transcripts per million mapped fragments (FPKM) of genes involved in encoding SOD **(B)**, CAT **(C)**, and POD **(D)**. **(E–G)** Change in the relative specific activities of SOD **(E)**, CAT **(F)**, and POD **(G)** in *M. micrantha* germs exposed to cold stress. One-way ANOVA, *p* ≤ 0.05 (*).

A total of 12 enzymes mainly involved in oxidation-reduction regulation were identified, including catalase (CAT), acyl-CoA oxidase, enoyl-CoA hydratase, peroxidase (POD), SOD, 3-hydroxyacyl-CoA dehydrogenase, glutathione peroxidase, adenylyl-sulfate reductase (glutathione), primary-amine oxidase, aldehyde dehydrogenase (NAD +), UDP-sulfoquinovose synthase, and adenylyl-sulfate reductase (Figure 5A). Among these, four SOD genes were highly expressed in germs subjected to 14°C temperature, indicating that a large amount of ROS is produced under cold stress. SOD usually operates as the first line of defense against ROS, while the associated scavenging pathways are complex and are strengthened under cold stress (Wang et al., 2019a). Furthermore, a total of seven CAT genes were upregulated at 14°C, indicating that CAT functions as the main ROS scavenging enzyme in plants. In addition, 53 POD genes were significantly upregulated at 14°C. Cold treatment induced a dramatic increase in POD activity at the beginning of seed germination, which was followed by an irreversible reduction in seed viability under the prolonged exposure to low temperature, resulting in constantly low POD activity. This result is consistent with the previously reported changes in POD activity during seed germination (Kaur et al., 2009; Wang et al., 2016).

The expression levels of *SOD* and *CAT* genes were significantly higher in germs that emerged at 14°C than in those that emerged at 25 and 30°C (Figures 5B,C). In addition, expression levels of genes encoding POD were remarkably lower at 14°C than at 25°C (Figure 5D). To further understand the changes in ROS levels, the activities of antioxidant enzymes were analyzed. The specific activity of SOD in germs at 30°C decreased by 52.74 and 57.38% at the 3^rd^ and 7^th^ days, respectively, compared with that at 25°C. Cold treatment at 14°C significantly increased the SOD activity by 54.26 and 59.27% at the 7^th^ and 14^th^ days, respectively, in comparison with the SOD activity at 25°C (Figure 5E). This trend is in line with the changes in protein and transcript levels, as shown in Figures 5A,B. Compared with 25°C, the activity of CAT increased significantly in *M. micrantha* germs germinated at 14°C, and the increase reached the highest level on the 10^th^ day in the cold-treated group (Figure 5F). On the 7^th^ and 10^th^ days of germination, the activity of CAT in germs at 14°C was markedly higher (by 41.77 and 50.27%, respectively) than that in germs at 25°C. Genes encoding CAT also showed coherent expression patterns (Figure 5A). It is possible that H_2_O_2_ concentration in *M. micrantha* germs under heat stress (30°C) is not high enough to stimulate CAT activity, because of the presence of other efficient scavengers potentially induced or stimulated by the stress conditions. A similar response of CAT to cold stress had been described in *Arabidopsis* (Zhong and McClung, 1996). In addition, under cold stress, the specific activity of POD in *M. micrantha* germs decreased significantly from the 7^th^ day onward at 25°C (*Q*-value = 0.0323) and declined steeply from the 10^th^ day onward at 14°C (Figure 5G), which is consistent with the average transcript levels of 118 POD-encoding genes (Figure 5D).

### DNA Methylome Profiling During *Mikania micrantha* Seed Development

**FIGURE 6:**
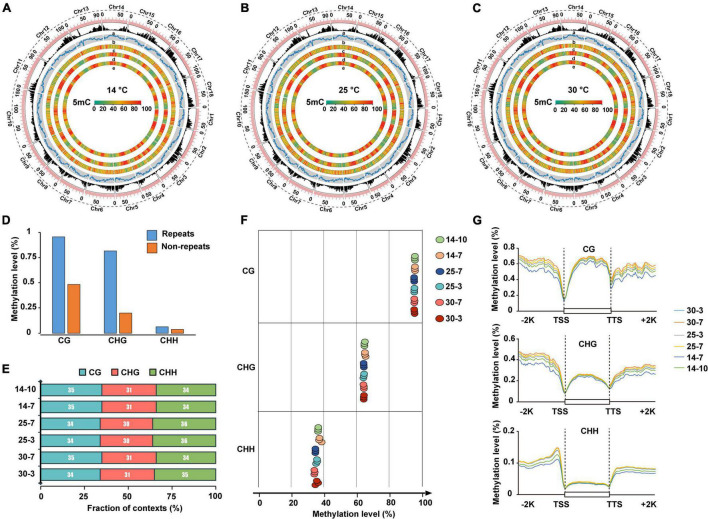
Whole-genome DNA methylation profiling during *M. micrantha* seed development. Genome-wide profiles of CG, CHG, and CHH DNA methylation in circos across the chromosomes of the *M. micrantha* seeds germinated at 14 **(A)**, 25 **(B)**, and 30°C **(C)**. **(a)** Gene density; **(b)** TE density; **(c–e)** methylation levels of CG, CHG, and CHH sequences contexts, from outer to inner rings. **(D)** DNA methylation levels of the repetitive sequence regions and non-repeat regions in the *M. micrantha* genome. **(E)** Proportion of CG, CHG, and CHH contexts in the total number of methylation sites of the germs collected from three temperatures. **(F)** Weighted methylation levels of CG, CHG, and CHH contexts of *M. micrantha* seeds germinated at 14, 25, and 30°C. **(G)** Regional methylation levels in three sequence contexts of *M. micrantha* germs across gene-body and flanking regions in samples. A 2-kb region of the upstream or downstream regions, as well as the gene-body regions, was appropriately divided into 100 equal bins for the calculation of the mean methylation level. TSS, the transcription starts site of genes: the first base transcribed at the 50 end of the gene; TTS, the location where transcription ends at the 30 end of a gene sequence; Promoter: a 2-kb region of the TSS upstream; other region: regions expected for TSS, TTS, intron, exon, and promoter.

To explore the epigenetic role of DNA methylation in cold stress tolerance of *M. micrantha* during seed germination, we performed bisulfite sequencing (BiSeq) analysis of the genomic DNA isolated from seeds at two successive stages of germination: seeds collected on the 3^rd^ and 7^th^ days of germination at 25 and 30°C; and seeds collected on the 7^th^ and 10^th^ days of germination at 14°C. Approximately, 954 Gb of high-quality sequencing data were generated, at an average sequencing depth of approximately 28.75 × per sample (Supplementary Table 4). In total, 81.33–92.84 million read pairs were uniquely mapped to the *M. micrantha* genome, with a unique mapping rate of 49.35–53.01%. To verify the efficiency of bisulfite conversion, unmethylated Lambda DNA was added to all samples as a control before bisulfite treatment. The average bisulfite non-conversion rate was calculated as approximately 0.26% in all samples, which is comparable with the corresponding rate previously reported in other species, such as tea plants (Li et al., 1996; Wulfridge et al., 2019; Tong et al., 2021). The results showed a high 5-methylcytosine (5-mC) level in the genomic regions of *M. micrantha* with high transposable element (TE) density and low levels of CHG and CHH methylation in gene-rich regions (Figure 6A). Similar methylation patterns were observed in seeds exposed to 25 and 30°C (Figures 6B,C). Repetitive regions showed significantly higher methylation levels compared with non-repetitive regions in the *M. micrantha* genome (Figure 6D). The 5-mC mark was found in the CG, CHG, and CHH contexts at all stages of seed development analyzed, accounting for 34, 31, and 35% of all methylated sites, respectively (Figure 6E). The CG context showed the highest level of DNA methylation (64.18%), followed by CHH (44.69%) and CHG (35.30%) contexts, a contrast to their methylation proportions (Figure 6F). The levels of DNA methylation in all three sequence contexts within the gene body and in flanking regions were also analyzed. At all stages of seed development, the CG context showed the highest methylation level within the gene body region, followed by CHG and CHH contexts (Figure 6G). The CG context methylation level within the gene body and flanking regions decreased with the reduction in temperature from 25 to 14°C. Furthermore, the level of DNA methylation in all three sequence contexts showed a sharp reduction in the region near the transcription start site (TSS) and the transcription termination site (TTS). The level of DNA methylation was the highest in upstream and downstream regions and the lowest in the gene body, especially in the CHG and CHH contexts. Compared with CG and CHG methylation, DNA methylation in the CHH sequence context displayed a declining trend from the flanking regions toward the gene body, suggesting the presence of a CHH island in regions flanking *M. micrantha* genes.

### Impact of DNA Methylation Dynamics on Gene Expression

**FIGURE 7:**
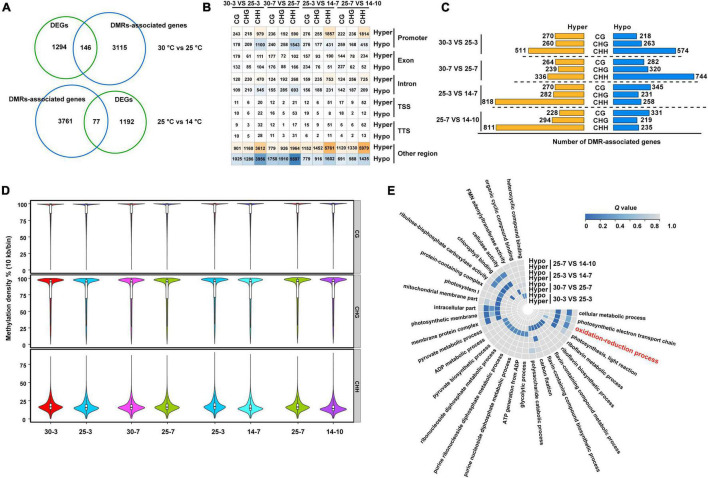
Diverse regulatory roles of DNA methylation on gene expression during *M. micrantha* seeds development. **(A)** Numbers of DMR-associated genes (a blue circle) and differentially expressed genes (a green circle) between successive stages of seed development are shown in the Venn diagrams. **(B)** A number of DEGs that are differentially methylated (hyper/hypo) in three sequence contexts (CG, CHG, and CHH) and genic regions between successive stages of seed development are given. Intensity of orange and blue indicates number of DMR-associated upregulated and downregulated genes, respectively. **(C)** Numbers of DMRs-associated genes identified from four comparisons selected stages of seed development (3^rd^ day at 30 and 25°C; 7^th^ day at 30 and 25°C; 3^rd^ day at 25°C and 7^th^ day at 14°C; 7^th^ day at 25°C and 10^th^ day at 14°C) are given in hyper/hypo-methylated with different sequence contexts *via* bar graphs. **(D)** A methylation level at individual mC in different sequence contexts at both stages of seed development at three temperatures is shown *via* boxplot. **(E)** Gene ontology (GO) analysis of DMR-associated genes (hyper/hypo methylated) at four comparisons is shown *via* a heatmap. The scale represents the *Q*-value of enriched GO terms.

Considering the association between DNA methylation and gene expression, we used RNA-Seq data to analyze the expression profiles of the differentially methylated region (DMR)-associated genes at different stages of seed development. As indicated in Figure 7A, a total of 4,530 DMR-associated (hypermethylated/hypomethylated) genes were identified during *M. micrantha* seed development. Among these, 146 and 77 DMR-associated genes exhibited different expression levels during 30– 25°C and 25–14°C transitions, respectively. A fraction of genes showed differential methylation (hyper/hypo) in different sequence contexts located in genic regions (Figure 7B).

Specifically, in the 30°C vs. 25°C comparisons, 979 and 590 genes were found to be hypermethylated on the 3^rd^ and 7^th^ days, respectively. Additionally, 1,100 and 1,543 genes were determined to be hypomethylated in the 30°C vs. 25°C comparisons. The hyper- or hypomethylation of DMR-associated genes mainly occurred in the protomer region, followed by the intron region. Most of the differential methylation was represented by hypermethylation in the CHH context during all successive stage transitions in both 30°C vs. 25°C and 25°C vs. 14°C comparisons (Figure 7C). Germs that emerged at 14°C were more likely to possess downstream CHH methylation than those that emerged at 25°C, indicating that cold stress leads to the initiation of the demethylation program during the development of *M. micrantha* germs (Figure 7D). GO enrichment analysis revealed differential methylation levels among genes involved in the photosynthetic electron transport chain, the oxidation-reduction process, photosynthesis, light reaction, and membrane protein complex (Figure 7E). Notably, irrespective of the sequence context and seed development stage, the level of DNA methylation at gene ends was low, probably to avoid methylation at or around the TSS.

**FIGURE 8:**
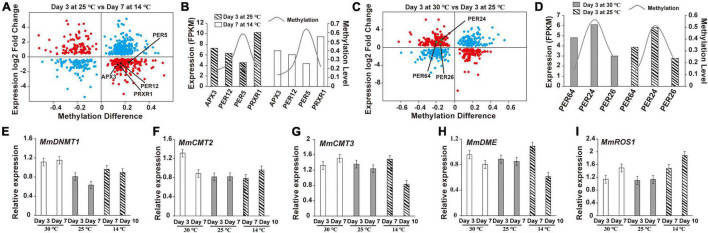
Expression and methylation patterns of cold-responsive genes during the germination of *M. micrantha* seeds. **(A)** Correlations between gene expression and methylation expression levels in the comparison of the 3^rd^ day at 25°C and the 7^th^ day at 14°C. X-axis represents the DNA methylation difference, and y-axis represents the genes expression log2 fold change. The blue plot indicates no association; the red plot means there exists an association. **(B)** Changing tendency between a methylation level and FPKM of genes identified from the comparison of the 3^rd^ day at 25°C and the 7^th^ day at 14°C. **(C)** Correlations between gene expression and methylation expression levels between the 3^rd^ day at 30 and 25°C. **(D)** Changing tendency between the methylation level and FPKM of genes identified from the comparison of the 3^rd^ day at 25 and 30°C. Relative expression of methyltransferase genes **(E–G)** and demethylation-related genes **(H,I)** performed using qRT-PCR.

To explore how DNA methylation and gene expression diverge during germ development, we divided genes into four groups depending on their expression levels (high, medium, low, and none), and then calculated the divergence between their DNA methylation and expression. The *Q*-values of all comparisons between DNA methylation and gene expression levels are summarized in Supplementary Table 5. Genes with high DNA methylation levels in all three sequence contexts in the upstream regions were highly expressed (Supplementary Figures 3–5), whereas those with high gene-body DNA methylation levels in CHG and CHH cytosine contexts exhibited low expression levels (Supplementary Figures 4, 5). Similar to the aforementioned discoveries, genes with high-level CHH methylation in downstream regions were expressed to higher levels than those with low-level CHH methylation. Although most of the DNA methylation was negatively correlated with gene expression during the development of *M. micrantha* germs, DNA methylation of certain cytosine contexts enabled the positive regulation of gene expression. This largely depended on the genic region and sequence context. Interestingly, 441 out of 1,002 DMR-associated genes showed an association between their FPKM values and DNA methylation levels on the 3^rd^ day at 25°C and on the 7^th^ day at 14°C (Figure 8A). One APX3- and three POD-encoding genes (*PER12*, *PER5*, and *PRXR1*) showed opposite trends between their expression and methylation levels (Figure 8B). Similarly, the expression of 395 genes was proven to be associated with their methylation divergence (Figure 8C). Three POD-encoding genes (*PER64*, *PER24*, and *PER26*) were identified in the 30°C vs. 25°C comparison on the 3^rd^ day, and a positive correlation between DNA methylation divergence and gene expression was observed (Figure 8D). The expression level of *MmDNMT1* presented an increasing trend at 14°C when compared to 25°C (Figure 8E). While another DNA methyltransferase gene *MmCMT2* showed no difference in gene expression can be registered between seed germination at the 3^rd^ day/25°C and the 7^th^ day/14°C, and a slight increase from the 10^th^ day at 14°C (Figure 8F). As a major factor in maintaining DNA methylation, although the expression level of *MmCMT3* was higher after 7 days of cold treatment, it showed a slight reduction to the 10^th^ day, indicating that cold stress interfered with the programmed methylation dynamics inside the *M. micrantha* germ by breaking methylation maintenance (Figure 8G). As concerns, the expression pattern of demethylation genes during cold treatment, expression levels of *MmDME* and *MmROS1* were enhanced on the 7^th^ day, and the relative expression of *MmROS1* reached 1.88 on the 10^th^ day under cold stress (Figures 8H,I), indicating that DNA methylation plays critical roles in the early response of the *M. micrantha* germ to cold stress, while the expression level of *MmDME* showed a decline at the 10^th^ day/14°C with no significant difference, which indicated that the demethylation process induced by cold stress diminished gradually with the germination proceeded.

### Real-Time Quantitative PCR Analysis of Genes Associated With Methylation Changes and Oxidoreductase Activity

A total of 30 methylation-related genes were identified in the *M. micrantha* genome (Supplementary Table 6). The results of qRT-PCR indicated that most genes responsible for *de novo* methylation *via* the RNA-directed mechanism (RdDM) pathway, especially those involved in Pol IV-dependent short-interfering RNA (siRNA) biogenesis, such as *MmDCL3*, *MmDCL4*, and *MmSHH1*, were highly expressed in the *M. micrantha* germ. This phenomenon suggests that the DNA methylation pathway is conserved in the *M. micrantha* germ during the whole germination process under cold conditions. Details of genes encoding DNA methyltransferases and demethylases are shown in Supplementary Table 7. To further validate the role of DNA methylation in the cold stress response, we examined the expression profiles of 10 randomly selected DNA methylation-associated genes by qRT-PCR. At 14°C, all genes involved in *de novo* methylation *via* the RdDM pathway showed similar decreasing expression patterns compared with their expression at 25°C (Supplementary Figures 6A–G). In addition, genes involved in the nucleosome remodeling and histone modification showed no change in the expression level at the three germination temperatures (Supplementary Figures 6H–J).

Twelve antioxidant genes exhibited differential expression patterns between the *M. micrantha* germ germinated at 25°C and that germinated at 14°C. The expression of CAT-encoding genes, *MmCAT1*, and *MmCAT2* was upregulated at 30°C, but a gradual reduction in expression was observed from 30 to 25°C (Supplementary Figures 7A,B). No difference in gene expression was detected between germination at 25 and 14°C (Supplementary Figures 7C,D). Thus, the expression profiles of selected *CAT* genes did not explain the progressive increase in CAT activity during germ development. All *SOD* genes, except *MmSOD3*, were upregulated as the temperature decreased, which is consistent with the change in SOD activity (Supplementary Figure 8). In addition, as shown in Supplementary Figure 9, only the decline in *MmPOD2* expression was consistent with the gradual reduction in POD activity under cold stress; gene expression profiles of the other *POD* genes did not explain the progressive increase in POD activity.

## Discussion

Invasive species are confronted with recurring environmental stresses throughout their life cycle, and seed germination is usually considered as the most sensitive developmental phase (Wang et al., 2015; Li H. et al., 2020). Cold stress is usually considered to be the greatest environmental threat affecting seed germination and seedling establishment (Chinnusamy et al., 2010; Gulshan et al., 2016). Here, we used DIA-MS, RNA-Seq, and genome-wide BiSeq approaches to explore the response of *M. micrantha* seeds to cold stress during germination. Compared with 25 and 30°C, the process of germination and germ growth was slow, and the rate of germination was reduced at 14°C, which illustrates that cold stress impedes the germination of *M. micrantha* seeds. The same results were obtained during the germination of *M. micrantha* seeds in soil culture (Supplementary Figure 2). Concomitantly, the physiological analysis showed that H_2_O_2_ and MDA contents and the conductivity of the seed leachate were increased at 14°C than at 25°C. This may be because of the reorganization of the cell membrane in *M. micrantha* seeds, which was severely destroyed when seed imbibition occurred under the cold condition, leading to the accumulation of MDA and oxides, especially H_2_O_2_, finally resulting in the delay or inhibition of seed germination. Oxidative stress caused by ROS, such as H_2_O_2_ and O_2_^–^, has been associated with the appearance of cold injury symptoms in many fruits, and is the primary factor involved in the modification of cell membrane conformation and structure under cold stress. Oxidative stress changes the lipid composition of the cell membrane, resulting in reduced permeability and fluidity. This phenomenon has been reported in many crop plants, including maize, cotton, and peanut (He et al., 2016; Gao et al., 2020; Chen et al., 2021). Moreover, the overaccumulation of H_2_O_2_ and O_2_^–^ leads to oxidative stress, which can strongly promote non-enzymatic lipid peroxidation, in which MDA is an important end product (Hodges et al., 1999; Sattler et al., 2006). The conductivity of the seed leachate may be an evidence of membrane damage induced by lipid peroxidation, since the MDA content of *M. micrantha* germs was higher at 14°C than at 25°C. Thus, it is clear that cold stress-induced cell membrane damage and ROS oxidative damage in *M. micrantha* seeds are the direct causes of seed germination inhibition at low temperatures.

Plant tolerance to cold stress largely depends on the reprogramming of gene expression, and transcriptional regulation is the key to cold acclimation (Chinnusamy et al., 2010). In this study, expression profiling of genes in *M. micrantha* germs germinated at 14°C revealed that genes related to catalytic activity, the cellular process, and antioxidant activity were mainly involved in the early response to cold stress to protect plant cells from freezing damage. This illustrates that the balance of intracellular oxygen metabolism was disturbed in *M. micrantha* seeds subjected to cold stress, which led to the accumulation of ROS. The ROS signals originated from different production sites within the plant cell have been reported to trigger dramatic transcriptional changes and cellular reprogramming, ultimately leading to programmed cell death (Petrov et al., 2015; Brown et al., 2020). To scavenge excess ROS, a rapid increase in SOD activity was observed in *M. micrantha* seeds during germination at 14°C from the 7^th^ day onward. Similarly, the activities of CAT enzymes were also significantly upregulated on the 10^th^ day at 14°C than at 25°C. The coordinated action of antioxidant enzymes is crucial for scavenging ROS to sense complex and variable temperature signals, and for regulating plant growth, development, and behavior to adapt to the environmental changes in temperature (Dai et al., 2009). In addition, morphological characterization of seeds using a stereomicroscope indicated that damage induced by cold stress to cell membranes was gradually alleviated as the germination was processed, and germ growth subsequently recovered to the normal level. Combining with the distribution loci of *M. micrantha*, we speculate that cold stress may be the main factor limiting *M. micrantha* seed germination in regions with temperature below 15°C, which might be an underlying reason for its inability to expand further to northern China.

Plants acquire freezing tolerance by modifying their transcriptome, proteome, and metabolome (Chinnusamy et al., 2010; Xu et al., 2019; Brown et al., 2020). DIA-based proteomic data showed that the reduction in temperature during germination had a strong effect on the total soluble protein categories in the *M. micrantha* germ (Figure 4D). Cold stress upregulated the capacity of ROS scavenging and redox adjustment by improving the activity of antioxidant enzymes, such as SOD and CAT during *M. micrantha* seed germination. Consistent with this observation, most of the DEPs responsible for oxidoreductase activity, response to oxidative stress, and the oxidation-reduction process were detected highly expressed in germs germinated at 14°C. This result is also in line with the measured antioxidant enzyme activities (Figure 5). Abiotic stresses, such as water shortage and low temperature, can activate the antioxidant enzymes activities for scavenging ROS in some species (Scebba et al., 2010; Wang et al., 2019b). In the current study, 12 enzymes involved in the redox process were upregulated among the DEPs identified from the 14°C vs. 25°C comparison. These enzymes included SOD, which is a ubiquitous enzyme in aerobic organisms and plays a key role in cellular defense mechanisms against ROS by modulating the amount of O_2_^–^ and H_2_O_2_ and decreasing the risk of OH^–^ radical formation (Bowler et al., 1992). We found that four SOD homologous proteins were highly expressed from the 7^th^ day to the 10^th^ day at 14°C, which may have reduced the cold-induced oxidative damage to plant cells. Interestingly, the expression of adenylyl-sulfate reductase (glutathione) was also enhanced. Under drought and cold stress conditions, adenylyl-sulfate reductase is responsible for ROS detoxification (Lu et al., 2019). The upregulation of adenylyl-sulfate reductase may help improve the *M. micrantha* germination rate and increase its root length, which also suggests that the rapid accumulation of antioxidants is an important determinant of *M. micrantha* germ cold tolerance.

Environmental factors modulate the epigenomic landscape and regulate adaptive responses in plants (Li R. et al., 2020; Zhang et al., 2021). Cytosine methylation (5-mC) is an epigenetic mark associated with developmental programs and stress response in plants, and DNA methylation can regulate the stress response in plants (Dowen et al., 2012; Liu et al., 2017). To investigate the epigenetic regulation of cold adaptation that inhibits *M. micrantha* seed development, we conducted a genome-wide analysis of changes in DNA methylation in germs of control plants (25 and 30°C) and cold-acclimated plants (14°C). A negative correlation was observed between DNA methylation in non-CG (CHG and CHH) contexts in the gene body and the expression levels of corresponding genes, suggesting the conservation of negative regulation of non-CG methylation in *M. micrantha* genes in the germ. No obvious consistent relationship was detected between gene expression and CG methylation in the gene body or downstream regions. These observations are similar to the findings in the tea plants, soybean, and chickpea (Liang et al., 2019; Wang et al., 2019a; Rajkumar et al., 2020). Generally, gene expression is negatively correlated with the DNA methylation level (Fray and Zhong, 2015; Xie et al., 2020). Nonetheless, our results showed that DNA methylation in *M. micrantha* germs did not always negatively regulate gene expression. CHH methylation in flanking regions and CHG methylation in upstream regions were observed to positively regulate gene expression. These results allow us to conclude that, although DNA methylation usually negatively regulates gene expression, flanking region CHH methylation and upstream CHG methylation can also positively regulate gene expression in *M. micrantha* germs. A likely explanation for this positive regulation is the presence of CHH islands in the flanking regions of *M. micrantha* genes in the germ (Gent et al., 2013; Fei-Man et al., 2018).

**FIGURE 9:**
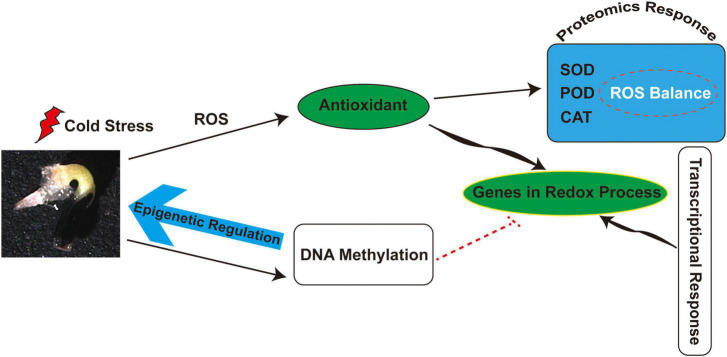
A schematic overview of the physiological and epigenetic response to cold stress during the seed germination of *M. micrantha.*

Several enzymes involved in *de novo* methylation and maintenance of methylation were also identified in *M. micrantha* germs, such as plant-specific chromomethylases (CMTs), which target CHH sites (CMT2) and CHG sites (CMT3). The phenomenon of *de novo* methylation in plants mainly depends on the RdDM pathway. DNA (cytosine-5)-methyltransferase 1 (MET1) maintains CG methylation, CHG cytosines are mainly methylated by CMT3, and, to a much lesser extent, by CMT2, while CHH methylation can be catalyzed only by DRM2 and CMT2 (Aufsatz et al., 2002; Zhang et al., 2018; Zhu et al., 2018). The slight decline of CMT2 and CMT3 expression in *M. micrantha* germs germinated at 14°C showed that non-CG methylations were progressively weakened under cold stress, which was consistent with lower CHG and CHH methylation levels detected by bisulfite sequencing. As suggested in Figure 9, cold stress leads to the accumulation of ROS and further stimulates antioxidant activity during *M. micrantha* seed germination. Proteomic and transcriptional analyses indicated that the enhancement of antioxidant activity within the *M. micrantha* germ maintains ROS homeostasis. Most importantly, DNA demethylation played an essential role in the *M. micrantha* seed germination through epigenetic regulation. In addition, we found that DNA methylation affected the expression of antioxidant genes to some extent.

## Data Availability Statement

The datasets presented in this study can be found in online repositories. The raw data of RNA sequencing, and DNA bisulfite libraries for this study have been submitted to the National Center for Biotechnology Information with accession number PRJNA766256. DIA proteomics sequencing datasets are available at iProX with Project ID IPX0003532000.

## Author Contributions

YS and TW conceived and designed the study. CC performed all experiments, contributed to data analysis, and finished the writing of this article. ZW checked the grammar. All authors reviewed and approved the final manuscript.

## Conflict of Interest

The authors declare that the research was conducted in the absence of any commercial or financial relationships that could be construed as a potential conflict of interest.

## Publisher’s Note

All claims expressed in this article are solely those of the authors and do not necessarily represent those of their affiliated organizations, or those of the publisher, the editors and the reviewers. Any product that may be evaluated in this article, or claim that may be made by its manufacturer, is not guaranteed or endorsed by the publisher.

## Footnotes

1
https://www.cvh.ac.cn/

2
http://www.nsii.org.cn/2017/home-en.php

3
https://www.cnki.net
