# Supplementary material for: Antioxidant Regulation and DNA Methylation Dynamics During Mikania micrantha Seed Germination Under Cold Stress
Source: Front Plant Sci. 2022 Apr 8;13:856527. doi: 10.3389/fpls.2022.856527 (PMC9024368; doi:10.3389/fpls.2022.856527)
Supplement: Supplementary file 8 [file Table_8.DOCX]

# Supplementary Information

# Transcriptome sequencing and data analysis

Germs germinated at 14 °C was performed as cold treat groups with 25 °C as controls, and 30 °C as heat treat groups. Total six groups of material were selected as the samples, and three biological replicates were set up for each group. Collected germs were ground with liquid nitrogen, total RNA was isolated from 200 mg germs using the RNAprep Pure Plant Kit (TianGen, Beijing, China) followed the manufacturer’s protocol. Nano Photometer® (Thermo Scientific, Wilmington, DE, USA) and Qubit2.0 Fluorometer (Life Technologies, CA, USA) were utilized to assess the purity and concentration of extracted total RNA. Then, 3 μg of total RNA from each sample was prepared to construct the cDNA library using the NEBNext®UltraTM RNA Library Prep Kit for Illumina (NEB). The mRNA was purified by Oligo (dT)-attached (Dynal, Life Technologies, CA, USA) magnetic beads from the total RNA, and then cut into pieces with fragment buffer at 20 °C. The first cDNA was reverse transcribed from RNA fragments with random hexamer as primer in the M-MuLV reverse transcriptase system. Polymerase chain reaction (PCR) was performed with Phusion High-Fidelity DNA polymerase, and library quality was assessed on an Agilent Bioanalyzer 2100 system. After pooled meeting the requirements for effective concentration and target data volume, the libraries were sequenced on the Illumina Hiseq 2000 platform (Beijing Novogene Bioinformatics Technology Co. Ltd). Trimmomatic version 0.35 was performed to trim raw reads for removing the adapter contamination and base calls in poor quality. Paired-end clean reads were aligned to the *M. micrantha* genome database (unpublished genome data from our laboratory) using HISAT2 v2.0.4. FPKM (expected number of fragments per kilobase of transcript sequence per million base pairs sequenced) was calculated using featureCounts v1.5.0-p3 based on gene length and read count. DESeq2 R package (1.16.1) was utilized to define the differential expression analysis of genes (DEGs) with adjusted Q value ≤ 0.05 and the absolute value of a fold change > 2.

**DIA-based proteomics analysis**

Data-dependent manner (DDA) analysis were carried out with a Q ExactiveTM HF-X (Thermo Fisher Scientific, PA, USA) with a Nanospray FlexTM (ESI) ion source. For library construction, peptides mixed with 0.4 μL iRT-standard peptides (Biognosys, Schlieren, Switzerland) were directly loaded onto a C18 Nano-Trap column (2 cm × 75 μm; 3 μm) (Thermo Fisher Scientific, PA, USA). For DIA LC-MS/MS analysis, the peptides were processed by tandem mass spectrometry (MS/MS) in EASY-nLCTM 1200 UHPLC system (Thermo Fisher Scientific, PA, USA) coupled to an Orbitrap Q Exactive HF-X mass spectrometer (Thermo Fisher Scientific, PA, USA). After being labelled, peptides were eluted at a 135-min gradient of solvent A (0.1% (v/v) formic acid in water) and solvent B (acetonitrile with 0.1% formic acid) using the following gradient: 0-40 min, 2% to 8% of B in 8 min. Peptides were separated using two mobile phases: solvent A (0.1% (v/v) formic acid in water) and solvent B (acetonitrile with 0.1% formic acid), with a 135-min gradient. Total fraction (1 μg) coupled with 0.4 μL standard peptides was analysed using the C18 Nano-Trap column (2 cm × 75 μm, 3 μm) (Thermo Fisher Scientific, PA, USA) in DIA mode.
