# Supplementary figures and images for: Antioxidant Regulation and DNA Methylation Dynamics During Mikania micrantha Seed Germination Under Cold Stress
Source: Front Plant Sci. 2022 Apr 8;13:856527. doi: 10.3389/fpls.2022.856527 (PMC9024368; doi:10.3389/fpls.2022.856527)

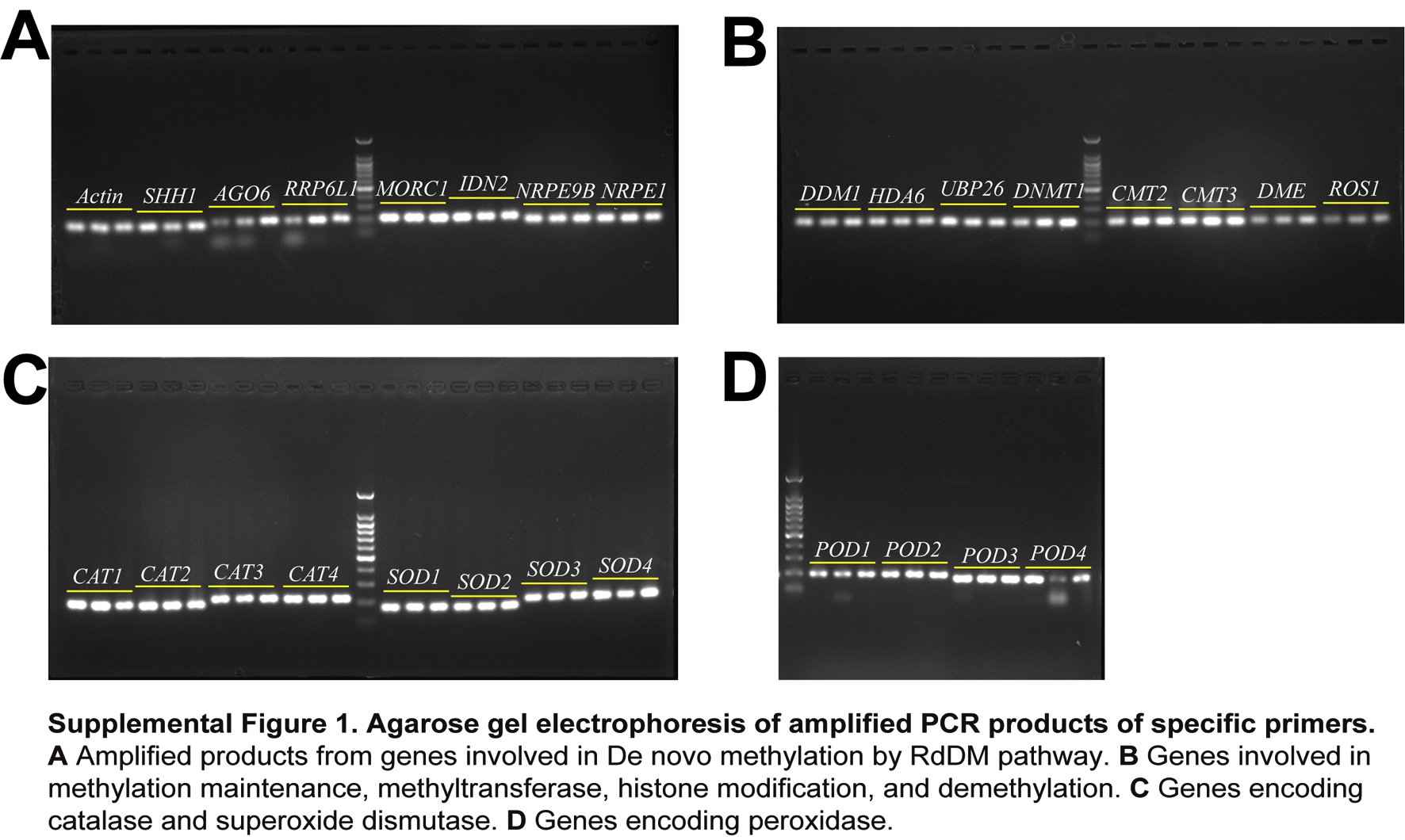

Supplement: Supplementary file 9 [file Image_1.TIF]

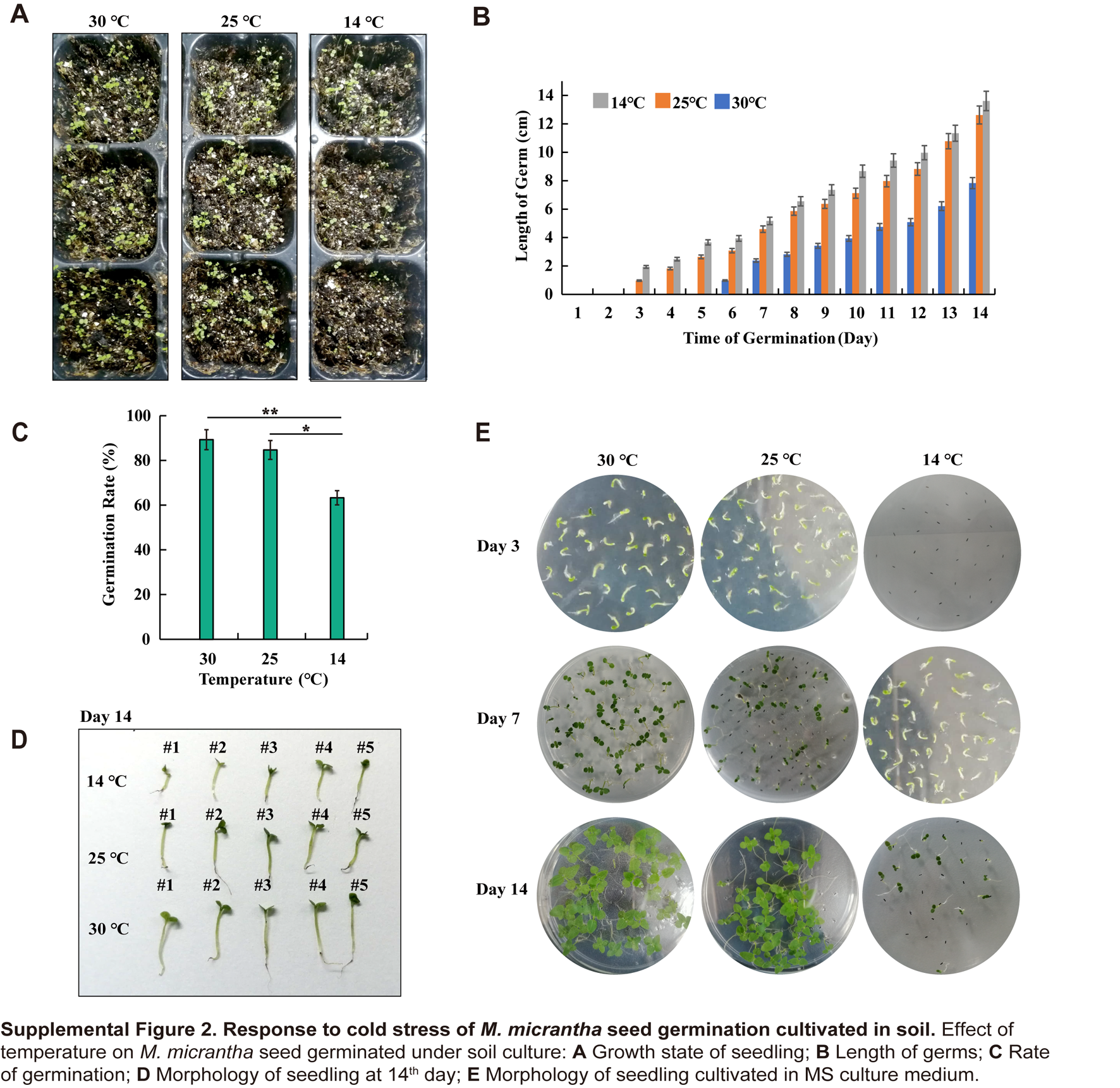

Supplement: Supplementary file 10 [file Image_2.TIF]

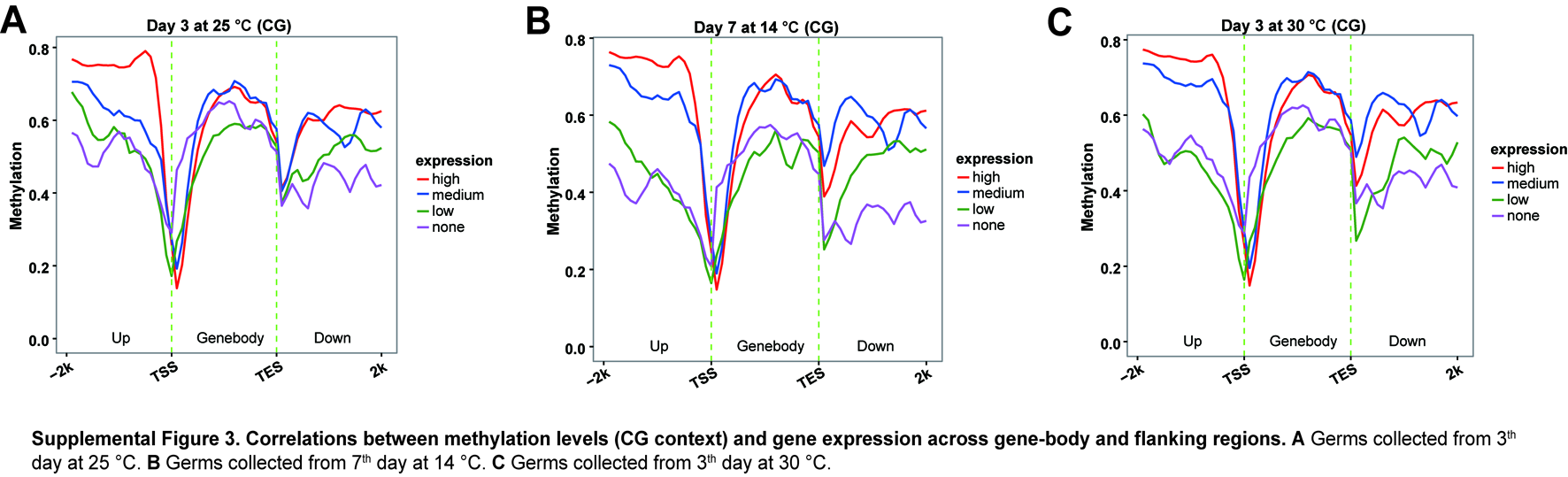

Supplement: Supplementary file 11 [file Image_3.TIF]

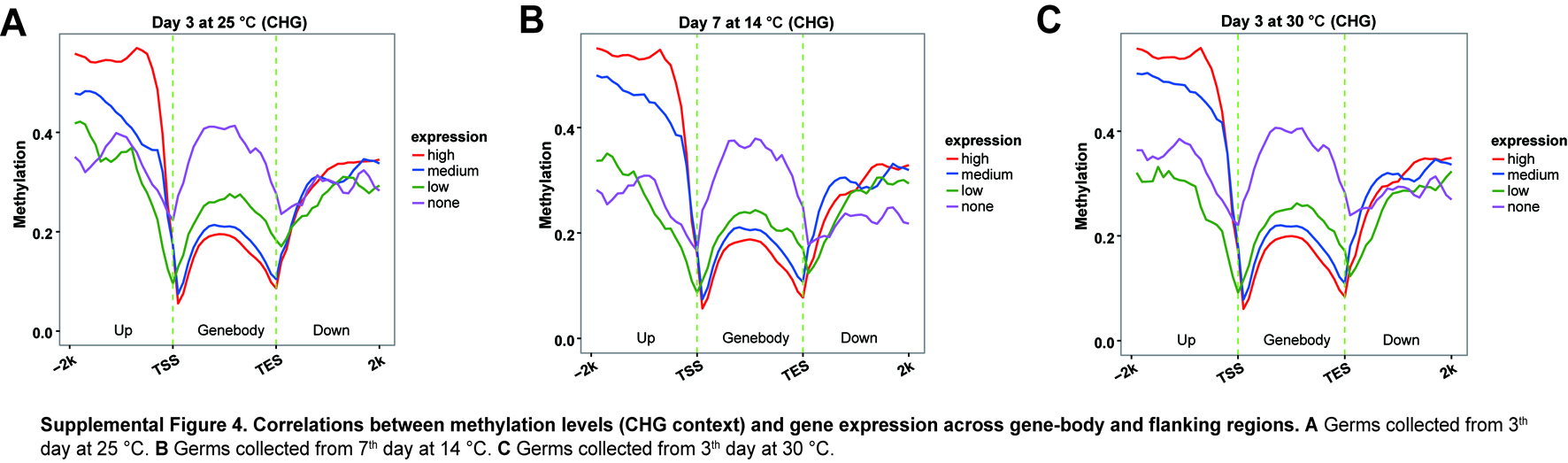

Supplement: Supplementary file 12 [file Image_4.TIF]

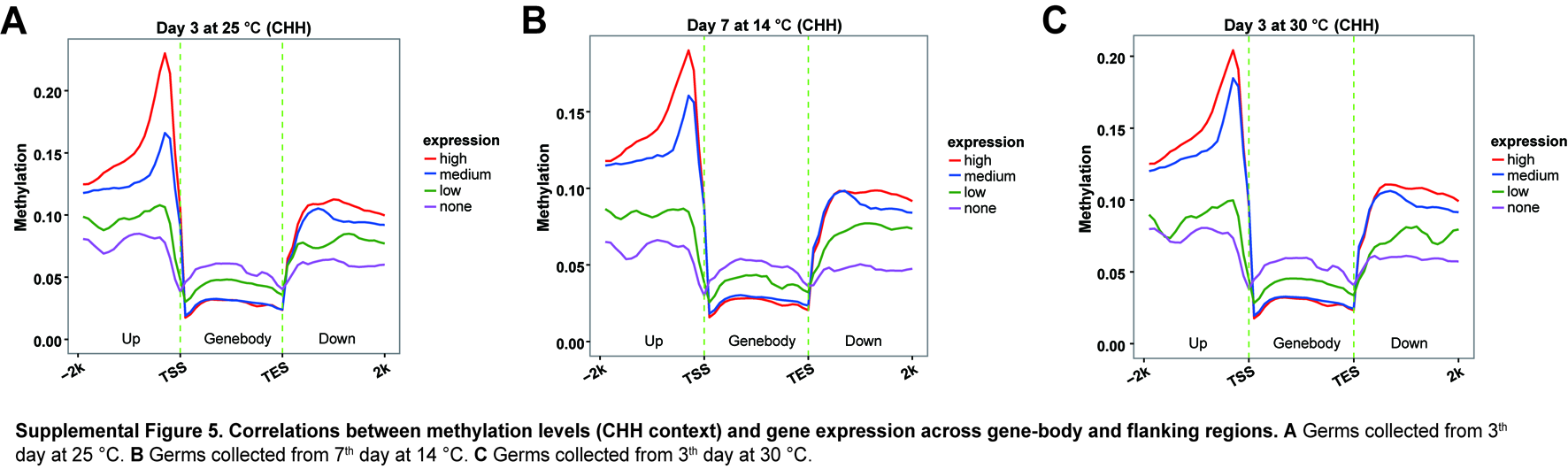

Supplement: Supplementary file 13 [file Image_5.TIF]

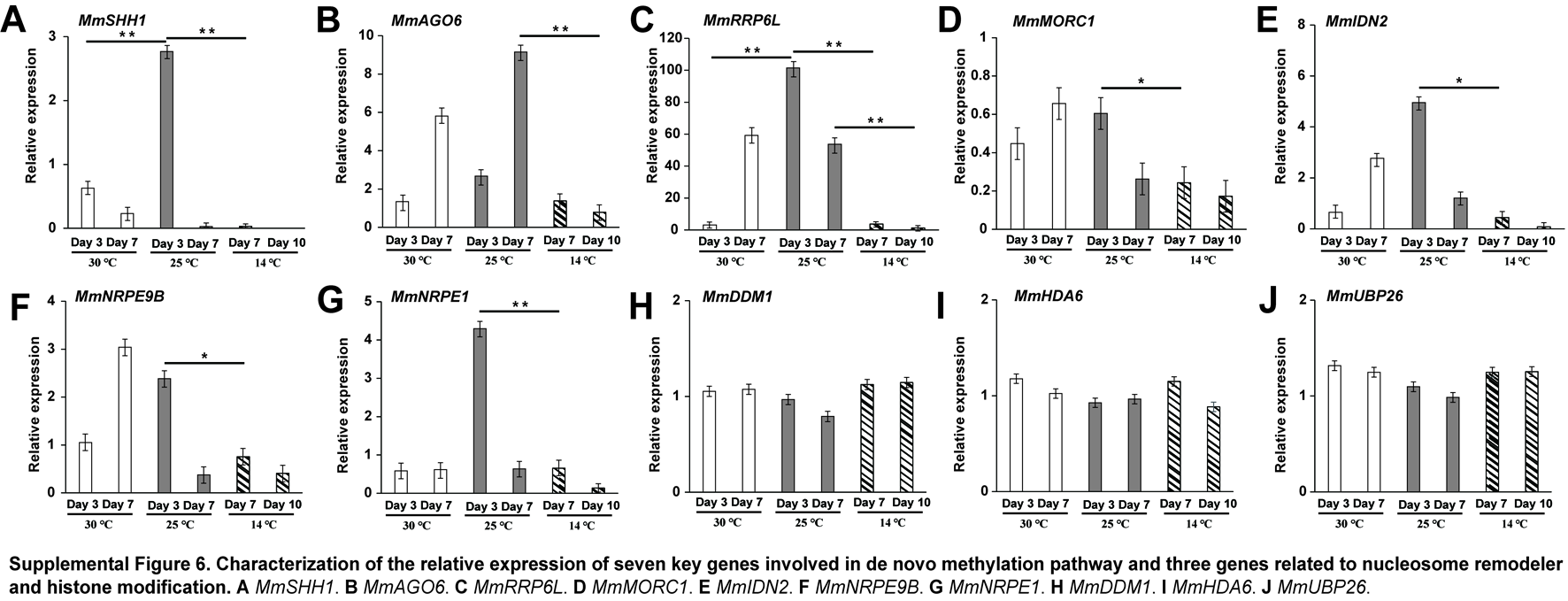

Supplement: Supplementary file 14 [file Image_6.TIF]

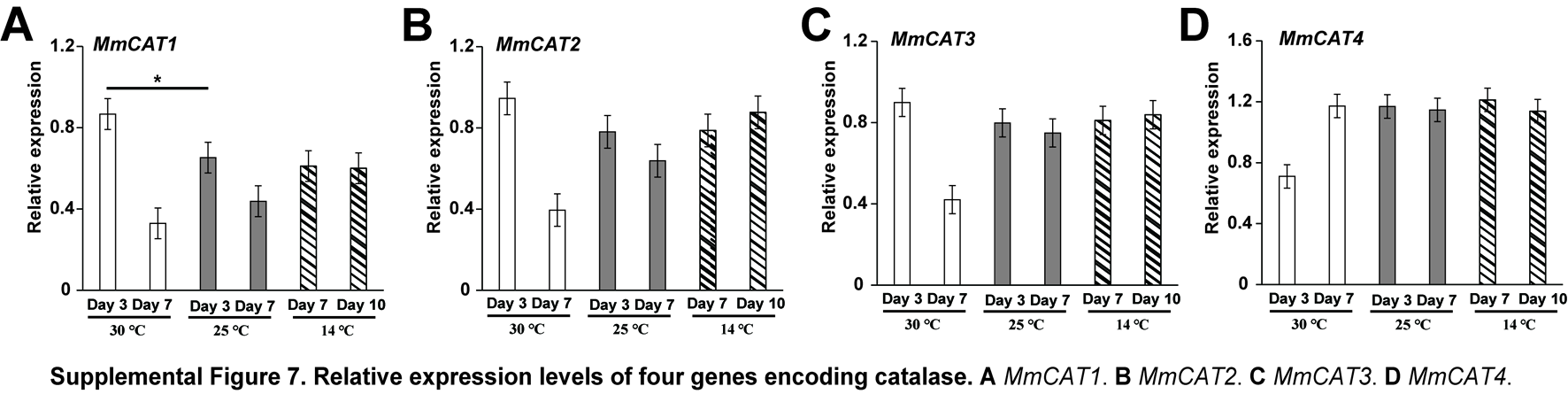

Supplement: Supplementary file 15 [file Image_7.TIF]

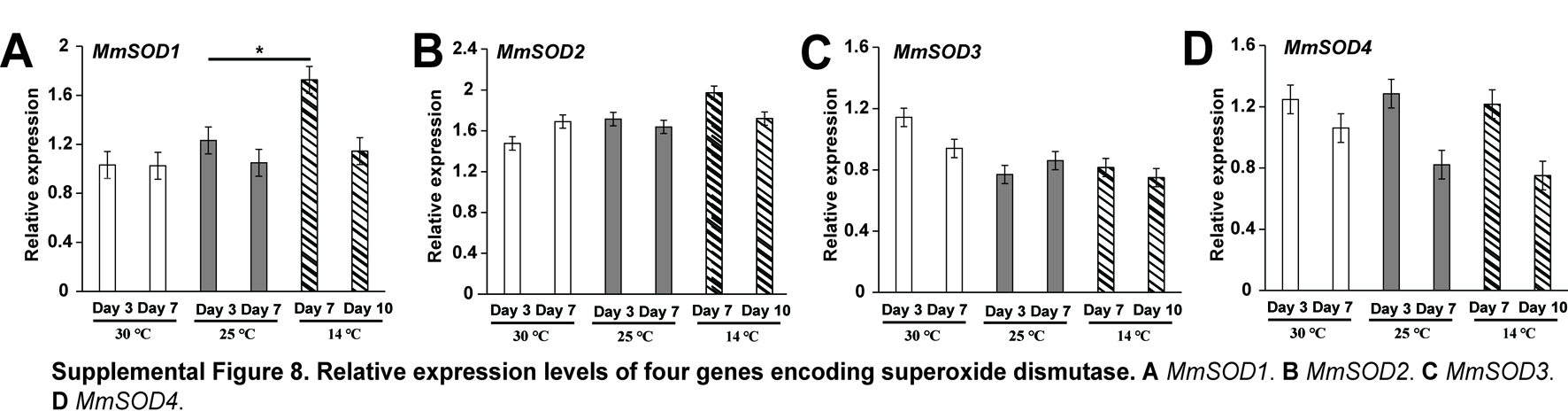

Supplement: Supplementary file 16 [file Image_8.TIF]

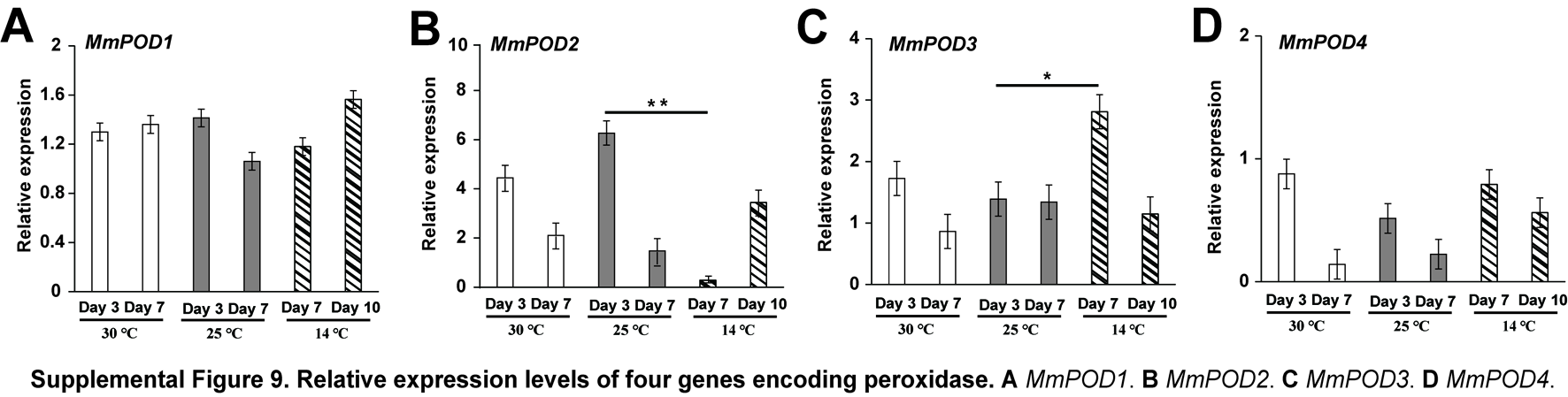

Supplement: Supplementary file 17 [file Image_9.TIF]
